# Phenotypic and genotypic survey of antibiotic resistance in *Salmonella enterica* isolates from dairy farms in Uruguay

**DOI:** 10.3389/fvets.2023.1055432

**Published:** 2023-03-09

**Authors:** María Laura Casaux, Bruno D'Alessandro, Rafael Vignoli, Martín Fraga

**Affiliations:** ^1^Plataforma de Investigación en Salud Animal, Instituto Nacional de Investigación Agropecuaria (INIA), Estación Experimental INIA La Estanzuela, Colonia, Uruguay; ^2^Departamento de Desarrollo Biotecnológico, Instituto de Higiene, Facultad de Medicina, Universidad de la República, Montevideo, Uruguay; ^3^Departamento de Bacteriología y Virología, Instituto de Higiene, Facultad de Medicina, Universidad de la República, Montevideo, Uruguay

**Keywords:** bovine salmonellosis, antimicrobial resistance, calves, WGS, genotyping

## Abstract

*Salmonella enterica* is an important zoonotic pathogen that is frequently identified in dairy farming systems. An increase in antibiotic resistance has led to inadequate results of treatments, with impacts on animal and human health. Here, the phenotypic and genotypic susceptibility patterns of *Salmonella* isolates from dairy cattle and dairy farm environments were evaluated and compared. A collection of 75 *S. enterica* isolates were evaluated, and their phenotypic susceptibility was determined. For genotypic characterization, the whole genomes of the isolates were sequenced, and geno-serotypes, sequence types (STs) and core-genome-sequence types were determined using the EnteroBase pipeline. To characterize antibiotic resistance genes and gene mutations, tools from the Center for Genomic Epidemiology were used. *Salmonella* Dublin (SDu), *S*. Typhimurium (STy), *S*. Anatum (SAn), *S*. Newport (SNe), *S*. Agona (Sag), *S*. Montevideo (SMo) and IIIb 61:i:z53 were included in the collection. A single sequence type was detected per serovar. Phenotypic non-susceptibility to streptomycin and tetracycline was very frequent in the collection, and high non-susceptibility to ciprofloxacin was also observed. Multidrug resistance (MDR) was observed in 42 isolates (56.0%), with SAn and STy presenting higher MDR than the other serovars, showing non-susceptibility to up to 6 groups of antibiotics. Genomic analysis revealed the presence of 21 genes associated with antimicrobial resistance (AMR) in *Salmonella* isolates. More than 60% of the isolates carried some gene associated with resistance to aminoglycosides and tetracyclines. Only one gene associated with beta-lactam resistance was found, in seven isolates. Two different mutations were identified, *parC_T57S* and acrB_R717Q, which confer resistance to quinolones and azithromycin, respectively. The accuracy of predicting antimicrobial resistance phenotypes based on AMR genotypes was 83.7%. The genomic approach does not replace the phenotypic assay but offers valuable information for the survey of circulating antimicrobial resistance. This work represents one of the first studies evaluating phenotypic and genotypic AMR in *Salmonella* from dairy cattle in South America.

## 1. Introduction

*Salmonella enterica* subspecies *enterica* (from now *S. enterica*) is one of the main pathogens affecting humans and animals and can cause various symptoms and illnesses, ranging from self-limiting acute gastroenteritis to septicemia and death (1, 2). Humans can be affected by typhoidal and non-typhoidal *Salmonella*. Typhoidal serotypes include invasive *Salmonella* Typhi and Paratyphi, which cause enteric fever (3). On the other hand, non-typhoidal *Salmonella* can cause enteric or invasive salmonellosis and is a major cause of morbidity and mortality globally (4). Many *Salmonella* infections in humans are due to the consumption of contaminated food, including meat of bovine origin, since this is a potential reservoir of the pathogen (5). *Salmonella* is now considered to cause more than 1 million infections annually in the US and is among the leading causes of foodborne illness (6). Animals are affected by non-typhoid serotypes such as Abortusovis, Dublin, and Gallinarum, which are adapted to sheep, cattle and poultry hosts, respectively, as well as other ubiquitous serotypes such as *S*. Typhimurium and *S*. Enteritidis (7).

In cattle, non-typhoid serotypes are capable of causing systemic disease similar to typhoid fever in humans (8). Salmonellosis in these animals is characterized by a more frequent occurrence in intensive farming systems. The disease varies according to different factors, such as the serotype involved, and the age, physiological and immune status of the host (9). *Salmonella* Dublin is the serotype adapted to cattle and can cause septicemia and abortions. *Salmonella* Typhimurium is frequently identified in calving systems and, in addition to enteric disease, can cause invasive diseases (10, 11). Both serotypes can rapidly spread in a herd, causing mortality and presenting a challenge in disease management (9).

Antibiotics are one of the main tools for treating bacterial diseases. However, in recent decades, the increase in AMR has led to inadequate treatment results, driving the search for new therapeutic options (12). When treating salmonellosis, antibiotics are indicated when the risk of systemic infection is increased, as in immunocompromised persons (13). Ampicillin and trimethoprim-sulfamethoxazole are used for the treatment of bovine salmonellosis, and fluoroquinolones and β-lactams are used for human treatment (14). However, the effectiveness of these antibiotics has decreased, mainly due to antibiotic resistance, which is a trend that is increasing in this bacterial genus and others (15, 16).

Plasmids are circular or linear extrachromosomal genetic elements present in various organisms (17). *Salmonella* serovars cause infections in animals and humans and may carry plasmids conferring virulence and AMR with particular biological properties that are subsequently expressed in the host (18). Within plasmids, antibiotic resistance genes are generally located in transposons. In addition, integrons are involved in the recruitment and expression of antimicrobial resistance genes (19).

In bacteria of animal origin, AMR varies among regions of the world. In the USA, *S. enterica* of bovine origin commonly shows resistance to cephalosporins and quinolones (20), while in Canada, the most common AMR pattern is resistance to ampicillin, chloramphenicol, streptomycin, sulfamethoxazole and tetracycline (ACSSuT) (21). Additionally, in South America, the AMR *Salmonella* isolates obtained from calves differ among regions. In northeastern Brazil, the main type of resistance observed is to cefotaxime (22), whereas in southeastern Brazil, nalidixic acid and cefoxitin resistance is primarily observed to, with a high frequency of susceptibility among strains (23). In Uruguay, the main phenotypic resistance observed is to streptomycin, tetracyclines and ampicillin, with differences in susceptibility between serotypes, where Dublin is more susceptible to antibiotics than Typhimurium (24).

In recent years, the complete sequencing of microorganism genomes has made it possible to approach their study such that the information available about them from different approaches, such as clinical diagnosis, epidemiology and research, has increased considerably (25, 26). There are currently several bioinformatics tools for the analysis of complete genomes. For AMR investigation, there are specialized databases with information about resistance genes and mutations. These databases are generally free to access and are periodically updated (27). Although genome-wide analysis does not replace phenotypic methods of resistance analysis, there is a high degree of correlation between the two approaches, and genome-wide analysis is a useful complement for the identification of new mechanisms and relationships between isolates (28).

Studies characterizing bovine-derived *Salmonella* strains are scarce in our country. Hence, the objective of this work is to study the presence and distribution of resistance genes, plasmids and integrons using different bioinformatic evaluation systems in a diverse sample of *Salmonella* strains of bovine origin from Uruguay.

## 2. Materials and methods

### 2.1. Strain collection

The study was conducted with 75 strains of *S. enterica* obtained from 2016 to 2020 at the Plataforma de Investigación en Salud Animal—Instituto Nacional de Investigación Agropecuaria, La Estanzuela, Uruguay. The collection was represented by 75 isolates obtained from stool samples collected from 35 calves, 5 cows, and 1 heifer; 25 organ samples from autopsied animals; 5 samples from the dairy farm environment; 1 food sample; 1 udder swab sample from milking cows; 1 drinking water sample; and 1 bovine fetus autopsy sample. The fecal isolates came from 26 calves < 20 days old and 7 calves between 20 and 30 days old. There were also two isolates from calves aged 65 and 114 days. The cows from which *Salmonella* isolates were obtained from feces were between 2 and 4 years old, and the heifers were 1 year old. The calves had diarrhea in all cases, and the cows were pregnant. The calves from which samples of organs and fluids were obtained had ages of <10 days in 7 cases, between 11 and 20 days in 7 cases, between 21 and 35 days in 3 cases, between 45 and 60 days in 4 cases, and between 80 and 90 days in 3 cases (Supplementary Table 1).

### 2.2. *Salmonella* collection, maintenance and growth

For the isolation of *Salmonella* spp. from different samples collected, the procedures described by Casaux et al. (24) were used. The strains were conserved in 20% glycerol and stored at −80°C. For reuse, they were cultivated in trypticase soy agar at 37°C for 18–24 h.

### 2.3. Antimicrobial susceptibility testing

The antibiotic susceptibility test was performed using the Kirby Bauer disc diffusion method (29). The antibiotics evaluated were ampicillin (AMP, 10 μg), amoxicillin-clavulanic acid (AMC 20/10 μg), cefotaxime (CTX 30 μg), sulfamethoxazole/trimethoprim (SXT 23.75/1.25 μg), ciprofloxacin (CIP 5 μg), enrofloxacin (ENR 5 μg), chloramphenicol (C 30 μg), streptomycin (S 10 μg), gentamicin (CN 10 μg), tetracycline (TE 30 μg), nitrofurantoin (NF 300 μg), and azithromycin (AZT 15 μg). *E. coli* ATCC 25,922 and *E. coli* ATCC 35,218 were used for quality control. The results were interpreted according to Clinical and Laboratory Standards Institute (31), and to facilitate the description of the results, both resistant and intermediate results were classified as “not susceptible.” For enrofloxacin, the CLSI guidelines for bacteria isolated from animals were used (30) (Supplementary Table 1). In addition, multidrug-resistant isolates (MDR) were considered when the isolates showed non-susceptibility to 3 or more groups of antibiotics (32).

### 2.4. Whole-genome sequencing

The 75 isolates were sent to the sequencing facility of MicrobesNG (Birmingham, UK, http://www.microbesng.com) for whole-genome sequencing. Standard sequencing services were provided by the company. Genomic DNA libraries were prepared with the Nextera XT Library Prep Kit (Illumina, San Diego, USA) according to the manufacturer's protocol with the following modifications: input DNA was increased 2-fold, and the PCR elongation time was increased to 45 s. DNA quantification and library preparation were carried out on a Hamilton Microlab STAR automated liquid handling system (Hamilton Bonaduz AG, Switzerland). The pooled libraries were quantified using the Kapa Biosystems Library Quantification Kit for Illumina. Sequencing was performed with Illumina sequencers (HiSeq/NovaSeq) using a 250 bp paired-end protocol. Adapters were trimmed from the reads using Trimmomatic 0.30 with a sliding window quality cutoff of Q15 (33). *De novo* assembly was performed using SPAdes version 3.7 (34), and contigs were annotated using Prokka 1.11 (35).

### 2.5. Genoserotyping, MLST and core genome MLST

The genome sequences of the *Salmonella* collection were analyzed using the EnteroBase platform. The serotype was determined using the SISTR1 + SeqSero2 tools (36–38). The core-genome MLST V2 experimental scheme (cgMLST V2) was used to obtain the value of the sequence type (cgST), and the Achtman MLST scheme was used for the allelic profile and the sequence type (ST) (39) (Supplementary Table 1).

### 2.6. Antimicrobial resistance genes, mutations and plasmids

To identify AMR genes and to chromosomal point mutations, we used ResFinder and Point Finder 4.1 (40–42) from the Center for Genomic Epidemiology (CGE). For this analysis, we selected a threshold of 90% for the sequence ID and 80% for the minimum length coverage. Manual curation was performed in those cases where identification was not univocal and the gene sequence could contain a coding sequence disruption leading to a pseudogene. We also used the PlasmidFinder 2.1 (43) and IntFinder 1.0 (44) tools to identify plasmids and integrons. For PlasmidFinder, we used thresholds of 95 and 60% for identity and coverage, respectively, and for IntFinder, we used a threshold of 90% for both identity and coverage.

### 2.7. Correlation between phenotypic and genotypic resistance profiles

The phenotypic results of the antimicrobial susceptibility were used test as a reference and were correlated with the presence or absence of the resistance and/or mutation genes detected from the evaluation of the complete genome considering a resistant isolate. For each group of antibiotics, concordance was considered to exist for those results with a not susceptible phenotype for which at least one gene or mutation explained that finding, and concordance with phenotypic susceptibility was considered to exist when no corresponding gene or mutation was found. Sensitivity (Se), specificity (Sp), positive predictive values (PPVs), negative predictive values (NPVs), and accuracy were calculated according to Trevethan (45).

### 2.8. Phylogenetic analyses

The serovars SAn, SDu, Sty, and SNe were chosen to perform the phylogenetic analysis, as they were collected from more diverse sources and included more isolates for comparison. For each of these serovars, a phylogenetic tree was generated using different reference genomes: Anatum—USDA-ARS-USMARC-1728 (NCBI accession NZ_CP014664.1), Dublin—CT_02021853 (NCBI accession CP001144.1), Typhimurium—LT2 (NCBI accession NC_003197.2), and Newport 0007-33 (NCBI accession NZ_CP013685.1).

Snippy software (version 4.6.0) (Torsten Seemann. https://github.com/tseemann/snippy) was used to map the trimmed reads to the reference genome and obtain a whole-genome alignment. Snippy was run with the default parameters, and the snippy-core script was run to generate the whole-genome alignment file (.full.aln). This file was then used as the input for Gubbins software (version 3.1.6) to produce a new alignment, from which the regions of high polymorphism density (probable regions of recombination) were removed (46). Gubbins was used with the following options: –threads 8, –first_tree-biulder fasttree, –tree-builder raxmlng, and –first_model JC.

Whole-genome phylogenies were produced using RAxML-NG software (version 1.1.0) based on the filtered_polymorphic_sites files (obtained in the previous step). RAxML was used with the following options: –model GTR + G, –seed 3 and –bs-metric fbp (47). The branch support values were transferred to the best-scoring tree of each sample by the RAxML-NG pipeline. Bootstrapping converged after 50, 250, and 996 replicates for SDu, Sty, and SNe, respectively. For SAn, 1,000 replicates were performed, but convergence was not reached because of the very low divergence of the sequences.

Phylogenetic trees with annotations were generated using the online tool iTOL v5 (https://itol.embl.de/) (48). The final tree figures were edited with the software Inkscape 0.91 (https://inkscape.org/).

## 3. Results

### 3.1. Determination of serotypes by WGS

Seven serotypes were identified after analyzing the genomes of the 75 collected isolates. The greatest number of isolates (32) were classified as *S*. Typhimurium, 24 as *S*. Newport, 11 as *S*. Anatum, 6 as *S*. Dublin, 1 as *S*. Agona, 1 as *S*. Montevideo and 1 as IIIb 61:i:z53. Each serotype was represented by a single sequence type (ST). ST10, ST19, ST45, and ST64 were detected in all the isolates of *S*. Dublin, *S*. Typhimurium, *S*. Newport or *S*. Anatum, respectively. In addition, ST13 corresponded to *S*. Agona, ST138 corresponded to *S*. Montevideo and ST430 corresponded to the strain serotyped as IIIb 61:i:z53 were detected identified. Among a total of 31 *S*. Typhimurium genomes, 24 different cgMLSTs were distributed on 26 different farms. A farm with 24 isolates of *S*. Newport presented 9 different cgMLSTs. In *S*. Dublin, 6 different cgMLSTs were observed. In *S*. Anatum, 5 cgMLSTs were observed in 11 total genomes. cgMLSTs 261271, 260644, and 275391 were identified in IIIB 61:I:Z53, *S*. Agona and *S*. Montevideo, respectively (Supplementary Table 1).

### 3.2. Antimicrobial susceptibility testing

Most of the isolates showed non-susceptibility to quinolones, tetracyclines and aminoglycosides. Fifty-eight isolates were not susceptible to the second generation-quinolone ciprofloxacin (77.3%). In addition, in this group, 5 isolates (6.6%) were not susceptible to enrofloxacin. Fifty-eight isolates (67%) were not susceptible to tetracycline, 64 (85.3%) were not susceptible to streptomycin, and 7 were also not susceptible to gentamicin (9.3%).

Only nine isolates (12%) were not susceptible to beta-lactam ampicillin, and 4 (5.3%) were not susceptible to amoxicillin-clavulanic acid. Every isolate was susceptible only to cefotaxime, and only 3 (4%) of the isolates were not susceptible to trimethoprim/sulfamethoxazole. A similar situation was observed for chloramphenicol, to which only 1 (1.3%) of the strains was not susceptible. There were 15 (20%) isolates that were not susceptible to nitrofurantoin, and 7 (9.3%) were not susceptible to azithromycin.

Considering MDR, 42 isolates (56%) were MDR. Twenty-two isolates were not susceptible to three groups of antimicrobials (29.3%), 15 were not susceptible to 4 groups (20%), four were not susceptible to 5 groups (5.3%) and one was not susceptible to 6 groups of antimicrobials (1.3%). Three isolates of *S*. Anatum were MRD, as were 14 *S*. Newport isolates, 24 *S*. Typhimurium isolates, and the only IIIb 61:i:z53 isolate. The most frequent MDR phenotype was CIP-S-TE, observed in 15 isolates, followed by CIP-S-TE-NF, in 6 isolates, and S-TE-NF and CIP-S NF, in 2 isolates each (Supplementary Table 2).

### 3.3. Antibiotic resistance-related genes detected in *Salmonella* genomes

After using ResFinder, 21 genes that predict phenotypic resistance were detected. In addition to the presence of the *aac(6')-Iaa* gene in all 75 isolates, 50 (66.6%) presented the *aph(3”)-Ib* and *aph(6)-Id* genes. The *aph(3”)-Ia, aadA1, aadA2*, and a*adA17* genes, which also confer resistance to aminoglycosides, were detected at a low frequency. Only three genes that confer resistance to tetracyclines were identified. The most abundant of these genes was *tet*A, found in 48 (64%) isolates, followed by *tetB* and *tetM*. Sulfonamide resistance genes were observed in 29 (38.6%) isolates, all of which presented the *sul2* gene, and two of these isolates also presented the *sul1* gene. The only identified gene for beta-lactam resistance was the *blaTEM-1B* gene, detected in 7 isolates, and the only gene for quinolone resistance was the *qnrB19* gene, detected in 5 isolates. The *floR* and *cmlA1, fosA, qac, dfrA*, and *lnu* genes, which confer resistance to phenicols, fosfomycin, ammonium compounds, trimethoprim, and lincosamides, respectively, were detected in 4 or fewer isolates. According to serotype, the gene *tetA* was present in *S*. Typhimurium, *S*. Newport, *S*. Anatum and *S*. Agona. Only *S*. Typhimurium and *S*. Anatum presented the sul2 gene and the *bla-TEM1 gene*. Similarly, only *S*. Typhimurium and *S*. Dublin carried the *qnrB19* gene. Two mutations were detected using the PointFinder database. The *parC_T57S* chromosomal mutation, conferring resistance to quinolones, was detected in 38 (50.6%) isolates, including all strains of *S*. Anatum, *S*. Newport, *S*. Montevideo, *S*. Agona and the IIIB 61:I:Z53 isolate. This mutation was not present in the *S*. Typhimurium and *S*. Dublin isolates. The other detected mutation, *acrB_R717Q*, which confers resistance to azithromycin, was detected in one strain of *S*. Dublin (Supplementary Tables 1, 2).

### 3.4. Plasmid detection

Twelve different incompatibility (Inc) groups were detected in 70 strains. Forty-nine strains showed one Inc group, 17 strains showed two, and 4 strains presented three. Three strains, *S*. Newport, *S*. Agona and IIIB 61:I:Z53, did not present any of these groups.

All but five isolates carried at least one Inc group. The most frequent of these groups were IncFII(pHN7A8), IncFII(S), IncFIB(S), Col440I and IncI1. All *S*. Typhimurium isolates showed Inc groups, and the most frequent combination was Col440/IncFIB(S).

*Salmonella* Dublin strains presented either IncX1 or IncFII(S). In *S*. Anatum, the combination of Col440I/IncHI2A/IncQ1 plasmids was present in 1 strain. Two strains presented 2 plasmids in the combinations IncI1/IncI2 and IncI1/IncQ1. Eight strains presented the IncI1 plasmid. A single strain of the *S*. Newport serotype presented the plasmids IncFII(pHN7A8) and IncFIB(S), and 20 strains presented the plasmid IncFII(pHN7A8). *Salmonella* serovar Montevideo had a single IncI1 plasmid (Table 1).

**Table 1:** Incompatibility groups of plasmids distributed in different serovars.

	***S*. Typhimurium**	***S*. Dublin**	***S*. Anatum**	***S*. Newport**	***S*. Montevideo**	**Total**
IncFII	ND	ND	ND	20	ND	20
Col440I	12	ND	1	ND	ND	13
IncFIB	2	ND	ND	ND	ND	2
IncFIB(S)	15	ND	ND	1	ND	16
IncFIC(FII)	2	ND	ND	ND	ND	2
IncFII(S)	15	3	ND	ND	ND	18
IncHI2A	ND	ND	1	ND	ND	1
IncI1	1	ND	10	ND	1	12
IncI2	ND	ND	1	ND	ND	1
IncI2(Delta)	1	ND	ND	ND	ND	1
IncX1	ND	3	ND	ND	ND	3
IncQ1	3	ND	3	ND	ND	6

ND, not detected.

### 3.5. Plasmid localization of resistance genes and class 1 integrons

In 52/75 isolates, at least one plasmid incompatibility group and some antibiotic resistance genes were detected in the same contig (see the detailed results in Table 2).

**Table 2:** Resistance genes detected in plasmid incompatibility groups.

**Inc. Group**	**Size (Kb)**	**Resistance genes**	**No**
IncFII	85 kb	*tetA, aph (3“)-Ib, aph (7)-Id*	21
Criptic plasmid	8,4	*tetA, aph (3”)-Ib, aph (7)-Id, sul2*,	19
ColE1	2,8	*qnrB19*	4
IncFIB	51,8	*tetM, floR, intI-1, [dfrA12, aadA2, cmlA1, aadA1, and* Δ*qacL]*	2
IncFIB(S)/IncFII(S)/IncQ1	117/124	*tetB,aph (3“)-Ib, aph (7)-Id, sul2, and blaTEM-1b*	2
incQ	11	*tetA, aph (3”)-Ib, aph (6)-Id, and sul2*	2
IncQ	6,5	*aph(3”)-1b, aph(6)-Id, sul2, and aph(3')-Ia*	1
IncQ	80,4	*tetA, aph (3“)-Ib, aph (6)-Id, sul2, intI-1, [dfrA7, qacE1-1, sul-1]*	1
IncHI2A/IncQ	221	*tetA, aph (3”)-Ib, aph (6)-Id, sul2, intI-1, [dfrA7, qacE*1*-1, sul-1]*	1
IncFIB(S)/IncFII(S)/IncQ1	113,6	*aph(3”)-1b, aph(6)-Id, sul2, aph(3')-Ia, and blaTEM-1B*	1
incN	40	*tetA*	1
IncFIB(S)/IncFII (S)	97	*tetA, aph (3“)-Ib, aph (6)-Id, sul2, aadA17, aph(3')-Ia, and lnu(F)*	1
IncI1-I(Alpha)	69,5	*bla*_TEM − 1B_	1

The genes that are between rect parenthesis are contained in Class I integrons. Genes grouped in brackets correspond to those included in class 1 integrons.

The most frequently detected antibiotic resistance-related incompatibility group was IncFII, which was identified as an 85 kb contig in 21 isolates. This contig encoded the *tetA, aph(3“)-Ib* and *aph(6)-Id* genes, which confer resistance to tetracyclines and streptomycin. These 85 kb fragments were 98% identical to two plasmids available in the NCBI databases (accessions CP042245 and CP025329) from veterinary isolates in China.

Second, a small 8.4 kb non-conjugative cryptic plasmid was detected in 19 isolates that encoded *sul2, aph(3”)-Ib, aph(6)-Id*, and *tetA*, conferring resistance to sulfas, streptomycin, and tetracycline. This small plasmid without a defined incompatibility group is widely reported in databases such as CP023647 and CP049284.

**Figure 1:**
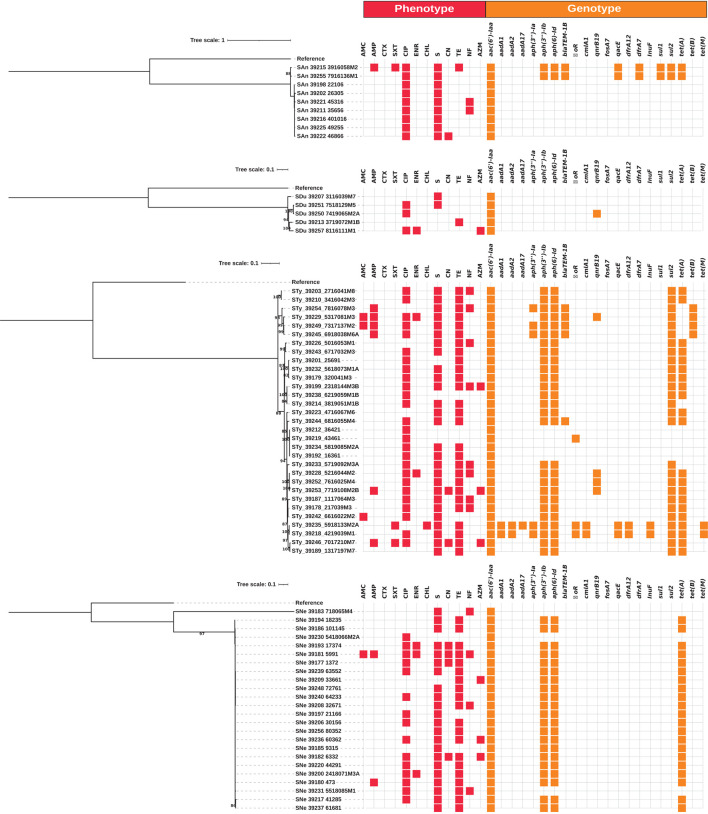
Whole-genome maximum likelihood phylogenetic trees (midpoint root) for the isolates from serovars Anatum (SAn), Dublin (SDu), Typhimurium (Sty) and Newport (SNe) in this study. Phenotypic antimicrobial resistance is indicated with red squares. Antibiotics: AMC, amoxicillin-clavulanic acid; AMP, ampicillin; CTX, cefotaxime; SXT, trimethoprim-sulfamethoxazole; CIP, ciprofloxacin; ENR, enrofloxacin; CHL, chloramphenicol; S, streptomycin; CN, gentamicin; TE, tetracycline; NF, nitrofurantoin; AZM, azithromycin. The presence of antimicrobial resistance genes is indicated with orange squares.

A third group of plasmids that can be highlighted in the context of the accumulation of resistance genes was IncQ. Although only 8 isolates presented IncQ, these non-conjugative plasmids were highly heterogeneous in terms of the resistance genes involved and their sizes and mode of presentation. Thus, in four isolates, IncQ was found as the only incompatibility group of the contig, while in another four, it was cointegrated with other incompatibility groups, such as IncHI2a, or even with the virulence plasmid of *Salmonella* spp. IncFIB(S)/IncFII(S). All detected gene arrangements can be seen in Figure 1.

Among the 75 isolates studied, only four carried class 1 integrons. Two highly related isolates of *S*. Typhimurium (39,218 and 39,235) presented the same integron on an IncFIB plasmid, with the arrangement intI-1, *dfrA12, aadA2, cmlA1, aadA1*, and *qacL*. Interestingly, isolate 39,235 but not 39,218 presented a second class 1 integron in the IncFIB(S)/IncFII(S) virulence plasmid encoding the aadA17 and *lnu(F)* genes.

On the other hand, two isolates of *S*. Anatum (39,215 and 39,255) carried the arrangement intI-1, *dfrA7, qacE*Δ*-1, sul1* in a contig associated with the cointegrated plasmid IncHI2A/IncQ1 or IncQ1, respectively.

### 3.6. Correlation between phenotypic and genotypic resistance

We recorded 900 phenotypic antibiotic susceptibility results and 1,575 genotypic data from CGE on 75 *S. enterica* isolates. The phenotype matched the genotype in 709 results. A total of 191 differences between phenotype and genotype were observed, 65 of which were due to the presence of phenotypic non-susceptibility with the absence of analyzed resistance genes/mutations, and 126 were due to phenotypic sensitivity but with the presence of resistance genes/mutations, with a general accuracy between the two tests of 83.6% (range 48–100%).

The highest correlation between phenotypic and genotypic resistance was found for gentamicin (100%), chloramphenicol (97.3%), trimethoprim-sulfamethoxazole (96%), macrolides (92%), tetracyclines (85.3%) and streptomycin for aminoglycosides (85.3%). The greatest number of discrepancies between the susceptibility profile and genes conferring resistance were observed in the of quinolone group (35 isolates), followed by the nitrofurantoin group (15 isolates). Among these three groups of antibiotics, the observed differences were due to the presence of non-susceptibility and the absence of genes and mutations in 40 isolates and 36 isolates with phenotypic susceptibility but also genes or mutations. According to the reference method, the overall sensitivity was 85.4% (14.3–100%), the overall specificity was 82.7% (38.8–100%), the PPV was 71.1% (11.6–100%), and the NVP was 90% (2–100%) (Table 3).

**Table 3:** Sensitivity and specificity of genotype predictions of resistant antimicrobial phenotypes for 75 *Salmonella* isolates.

**Antibiotics**	**Phenotype resistant/intermediate**		**Phenotype susceptible**		**Sensitivity % (a/(a + c)) × 100**	**Specificity % (d/(b + d)) × 100**	**PPV % (a/(a + b)) × 100**	**NPV % (d/(c + d)) × 100**	**Accuracy % ((a + d)/(a + b + c + d)) × 100**
	**Genotype resistant (a)**	**Genotype susceptible (c)**	**Genotype resistant (b)**	**Genotype susceptible (d)**					
**Aminoglycosides**									
STR	64	0	11	0	100	ND	85,3	ND	85,3
CN	7	0	0	0	100	ND	100	ND	100
**Tetracycline**									
TET	44	7	4	20	86.2	83.3	91.6	74.0	85.3
**Quinolones**									
CIP	29	28	11	7	50.8	38.8	72.5	20.0	48.0
ENR	5	1	38	31	83.3	44.9	11.6	96.8	48.0
**Penicillin**									
AMP	5	4	2	64	55.5	97	71.4	94.1	92
**Beta-lactam/beta-lactam inhibitor**									
AMC	2	2	5	66	100	93	28.6	97	90.6
**Cephems**									
CTX	0	0	7	68	ND	90.6	ND	100	90.6
**Trimetoprim-sulfametoxazol**									
SXT	2	1	2	70	66.6	97.2	50	98.5	96
**Macrolide**									
AZM	1	6	0	68	14.3	100	100	91.9	92
**Phenicol**									
CHL	1	0	2	72	100	97.2	100	100	97.3
**Nitrofuran**									
NF	0	15	0	60	ND	100	ND	80	80
Total					85.4	84.2	71.1	90	83.7

STR, streptomycin; CN, gentamicin; TET, tetracycline; CIP, ciprofloxacin; ENR, enrofloxacin; AMP, ampicillin; AMC, amoxicillin-clavulanic acid; CTX, cefotaxime; AZM, azithromycin; CHL, chloramphenicol; NF, nitrofurantoin; ND, not determined.

### 3.7. Phylogenetic analysis

Whole-genome phylogenetic trees were produced for strains of the serovars SAn, SDu, STy and SNe, as they were the most frequent serovars present on more than one farm and/or included more isolates for comparison. Independent phylogenetic trees were produced for each serovar, as this approach could resolve in more detail the intraspecific differences among isolates, and the results are shown in Figure 1.

In broad terms, while the observed genotypes were roughly the same among a given serotype, greater phenotypic diversity was observed for AMR. Strains from the same farm were clustered together in their respective trees.

The STy strains were the most genetically diverse group, in accordance with the broader range of sources and AMR observed for this serovar, as described above, whereas the SAn isolates were less diverse.

## 4. Discussion

*Salmonella enterica* is a pathogen present on dairy farms in Uruguay. In this study, 8 serotypes were identified, and each showed a single sequence type, suggesting the presence of a single lineage of strains.

As observed in Canada (21), *S*. Typhimurium ST19 was the most frequent sequence type isolated from calves with diarrhea, being identified 26 different times on 16 different dairy farms. This contrasts with findings in the USA, where the most frequent serotype is *S*. Dublin (49). ST19 has been identified in other countries and is associated with gastrointestinal disease due to showing a greater immune response to the flagellar antigens of these strains (50, 51). On one dairy farm, we identified 24 *S*. Newport ST45 isolates, most of which were susceptible to antibiotics. This ST affects both humans and animals, including cattle, and has been reported to show an MDR phenotype (52–54). In agreement with other reports, *S*. Dublin ST10 was isolated from 5 different farms, and *S*. Anatum ST64 was isolated from 2 farms (55–60).

*Salmonella* Agona is a pathogen frequently detected in food worldwide (61). In this work, the representative ST13 was isolated from a mesenteric lymph node. The isolate of *S*. Montevideo belonged to ST138, and this microorganism has also been reported as a foodborne pathogen (62). This sequence type of bovine origin is implicated in foodborne illness and comes from a different clade from those isolated from human outbreaks. Isolate IIIB 61:I:Z53 belongs to sequence type ST430, which has only been reported in association with human infections in the US due to contact with small turtles and their environment (63, 64). Here, it was isolated from the feces of a calf suffering from an outbreak of neonatal diarrhea and calf mortality, so its implications for farm animals should be evaluated.

We observed differences in the susceptibility profiles between the different serotypes. *Salmonella* Typhimurium was the serotype with the greatest differences in susceptibility. This could be explained by the diversity of geographic locations represented by the isolates since it was the most numerous serotype. In this study, the most frequent non-susceptibility pattern was found for aminoglycosides, tetracyclines and quinolones, identified in 11 (34%) isolates, 81% of which were MDR. A similar characteristic was observed in Canada by Otto et al. (21). Great differences are found among the reports of resistance in *S*. Dublin (21), and in our work, the isolates presented susceptibility to all antibiotics except aminoglycosides. The most frequent non-susceptibility pattern observed in *S*. Newport isolates, obtained from a single farm, was similar to that of *S*. Typhimurium, and this serovar is usually reported as MDR (65, 66), unlike *S*. Anatum, which was not susceptible to only aminoglycosides and quinolones.

According to the evaluation of the obtained genomes, we observed that the most frequent resistance genes in all serotypes were those that confer resistance to aminoglycosides, tetracyclines and sulfonamides. Resistance to aminoglycosides mediated by the *aac (6')-Iaa* and *aacC (6')-Iy* genes is not considered by relevant platforms because studies indicate that they do not confer resistance and are ubiquitous in *Salmonella* (67). However, the aac(6')-Iaa gene was detected in 100% of the strains in our study. The *aac(6')-Iaa* gene has been previously detected in human *Salmonella* isolates from Uruguay (68). It confers resistance to tobramycin, amikacin and kanamycin, but this gene is less efficient in the acetylation of gentamicin, which may explain the low rate of detection of phenotypic resistance in our results (69). The *aph(6)-Id, aph(3)-Ib* and *aph(3)-Ia* genes were detected less frequently. These last three genes were recently reported in *S*. Dublin isolated from bovines in the USA (59).

The most frequent tetracycline resistance gene identified was the *tetA* gene, which was present in those strains that did not present the *tet*B gene; both of these genes expulsion pumps in gram-negative bacteria (70). In previous reports, it was observed that the *tet*A gene presented a greater dissemination capacity than the *tet*B gene on farms (71). This finding could be explained by the fact that these genes occur in mobile genetic elements that are frequently conjugative (72).

The *sul*2 gene was detected at the highest frequency, followed by *sul*1, both of which confer resistance to sulfonamides. This contrasts with findings in other animal sources, where *sul1* is more prevalent (72, 73). This result was not confirmed phenotypically because we assayed trimethoprim and sulfamethoxazole antibiotics together. According to this combination, only 4 isolates carried *sul1/sul2* and *dfr* genes that confer resistance to trimethoprim-sulfamethoxazole.

Plasmid-mediated quinolone resistance (PMQR) generates a low level of resistance to quinolones but promotes the selection of other resistance mechanisms, and their rapid spread and has been reported in different bacterial species of animal origin (74–77). In this study, the *qnrB19* gene was detected in only 4 *S*. Typhimurium and 1 *S*. Dublin isolates. Similar isolates harboring this gene have been observed in South America (78–80). These results contrast with the published data from the region, where the most frequent variants are indicated to be *qnrB2, qnrS1*, and *qnrE1* (80).

In this work, we only detected a mutation in *par*C at position *57*, causing a *Thr* to *Ser* change in the topoisomerase that confers resistance to quinolones (80). Enrofloxacin is a representative antibiotic used in the veterinary industry, and due to its wide range of activity, it is frequently used for treating various clinical presentation (81, 82). The *parCT57S* mutation has been reported as the most frequent mutation in environmental samples and animal feces isolates in Brazil (80), and a single mutation in a topoisomerase IV or DNA gyrase gene confers high-level resistance to this group of drugs (75). In Uruguay, the *parCT57S* mutation and the presence of *qnrB19* were detected in human *S*. Typhimurium isolates between 2011 and 2013 (68). Taken together, our findings suggest that these genes and mutations have existed for a long time.

In the USA, the detection of the *blaTEM* gene is very frequent (59, 83, 84); in contrast, only 7 strains carried this gene in this work, corresponding to <10% of the collection. Following the results obtained from Resfinder, the *blaTEM-1* enzyme was only identified in 5 strains of *S*. Typhimurium and 2 strains of *S*. Anatum. Among these 7 isolates, only five showed reduced sensitivity to ampicillin and two to amoxicillin-clavulanic acid. Most of these *blaTEM-1*-carrying strains were isolated from neonatal calves.

We detected the *aadA1* and *aadA2* genes in two MDR *S*. Typhimurium isolates, and one of them even carried the *aadA*17 variant, which is frequently reported in England (85) but has not previously been reported in *Salmonella* isolates in South America. This class of genes is located within integrons (86), together with genes such as *sul, qacE*Δ1, *drf*, and *cmlA* that confer resistance to sulfonamides, antiseptics and disinfectants, trimethoprim and phenicol, respectively (73, 87–89). In our work, *aadA17* was also found within a class 1 integron but was only accompanied by the *lnu(F)* gene encoded by the virulence plasmid of *Salmonella* spp. Inc FIB(S)/IncFII(S).

On the other hand, two isolates of *S*. Anatum with similar characteristics were detected on the same farm but came from two calves sampled months apart. These strains presented *blaTEM, sul2, tetA, dfrA7, qac*Δ*1*, and *sul1*, among which the last three genes are part of a class 1 integron.

The *fosA* gene is widely distributed among Enterobacteriaceae (90), and the f*osA7* variant has been detected in plasmids in *S. enterica* from birds and in pigs and cattle in China (91, 92), conferring a high level of resistance to fosfomycin. In our study, this gene was detected in *S*. Agona and *S*. Montevideo isolated from mesenteric lymph nodes and feces, respectively. This gene was previously detected in Uruguay, but within *E. coli* STEC strains (93).

Interestingly, a single strain of *S*. Dublin had the *acrB_R717Q* mutation, which confers resistance to azithromycin, and was recently identified in Bangladesh in *S*. Typhi (94). Additionally, this mutation was recently reported in a study involving *S*. Dublin isolates of bovine origin by García-Soto et al. (95) in Germany.

In general, most plasmids were exclusive to a certain serotype. The plasmids shared by different serotypes were Col440I in *S*. Typhimurium and *S*. Anatum; IncFIB(S) in *S*. Typhimurium and *S*. Newport; IncI1 in *S*. Typhimurium, *S*. Anatum and Montevideo; and IncQ1 in *S*. Typhimurium and *S*. Anatum. This agrees with findings reported by Feng et al. (96), who indicated that serotypes generally do not share the same plasmids due to their mostly vertical inheritance. Previous studies reported that the most frequent plasmids detected in *S*. Dublin were IncX1, IncA/C2, and IncFII (59, 97). Although the number of *S*. Dublin strains evaluated in our study was very limited, the detected plasmids were similar, except for IncA/C2. While *S*. Typhimurium was the sole serovar harboring the plasmids IncA/C, IncI, Col, IncFII, IncFIB, and IncFIA (similar to the plasmids detected in our strains), there was a difference in the Newport serotype (in which the only detected plasmids shared with this work were the IncFII and IncFIB) and in Anatum and Agona (84). It is important to deepen the study of the plasmids present in the strains since they are used for epidemiological studies and the interpretation of the virulence and resistance observed in different regions.

Since 1960, the most commonly reported MDR phenotype in *Salmonella* has been resistance to ampicillin, chloramphenicol, streptomycin, sulfonamides, and tetracyclines (98). Most of the isolates were MDR, and 35.7% of these isolates showed the CIP-S-TE profile. Genotypically, we also observed MDR in 40% of the isolates, which carried resistance genes from 3 to 7 groups of antibiotics. Most of these MDR isolates were *S*. Anatum and *S*. Typhimurium.

Different publications have described notable correlations between resistance phenotypes and genotypes (83, 99). We detected a 100% correlation for gentamicin; above 90% for chloramphenicol, beta-lactams, trimethoprim-sulfamethoxazole and macrolides; and above 80% for tetracyclines, nitrofurans and streptomycin. Similar results were obtained by Campioni et al. (100) in *Salmonella* from different sources in Brazil.

In our work, although the estimated values of sensitivity, specificity, PPV and NPV were generally high, this was not observed in some specific drug groups (i.e., ciprofloxacin). With the strategy used in this work, we could not identify the presence of pseudogenes in the detected AMR genes that might account for part of this discrepancy. Other factors may be involved, such as low levels of expression or mutations in promoters or in Shine Dalgarno sequences. On the other hand, under the adopted strategy, we did not analyze the presence of efflux pumps since their effect depends on the level of expression. This aspect could explain certain levels of resistance that are not explained by our results.

Based on the above, phenotypic evaluation is still the most relevant approach when therapeutic decisions are being made.

This work represents one of the first studies in the region using the phenotypic resistance and WGS of *S. enterica* obtained from bovines as an analysis strategy. According to our results, *S*. Typhimurium was the serotype with the highest frequency and distribution, followed by *S*. Dublin, and all the serotypes detected were from a single sequence type. These serotypes were zoonotic, and we also found a high frequency of resistance to antibiotics used on farms and in human health. Taking these results into account, it is necessary to consider the implementation of strategies to limit the use of antibiotics that are considered critical and to mitigate the increase in resistance globally.

## Data availability statement

The data presented in the study are deposited in the EnteroBase (https://enterobase.warwick.ac.uk) repository, accession numbers SAL_HB5975AA, SAL_HB5235AA, SAL_HB5236AA, SAL_HB5237AA, SAL_HB5238AA, SAL_HB5239AA, SAL_HB4194AA, SAL_HB5240AA, SAL_HB5241AA, SAL_HB5242AA, SAL_HB5243AA, SAL_HB5299AA, SAL_HB5300AA, SAL_HB5301AA, SAL_HB5302AA, SAL_IB4224AA, SAL_HB5471AA, SAL_HB5474AA, SAL_HB5476AA, SAL_HB5740AA, SAL_IB4223AA, SAL_HB5741AA, SAL_HB5742AA, SAL_HB5743AA, SAL_HB5744AA, SAL_HB5745AA, SAL_HB5746AA, SAL_HB5747AA, SAL_HB5748AA, SAL_HB5749AA, SAL_IB4193AA, SAL_HB5976AA, SAL_HB5977AA, SAL_HB5978AA, SAL_HB5979AA, SAL_HB5980AA, SAL_HB5981AA, SAL_HB5982AA, SAL_HB5983AA, SAL_HB5984AA, SAL_HB5985AA, SAL_HB5986AA, SAL_HB5987AA, SAL_HB5988AA, SAL_IB4194AA, SAL_HB5989AA, SAL_HB5990AA, SAL_HB5992AA, SAL_HB5993AA, SAL_HB5994AA, SAL_HB5995AA, SAL_HB5996AA, SAL_HB5997AA, SAL_HB6171AA, SAL_HB6172AA, SAL_HB6173AA, SAL_HB6179AA, SAL_HB6188AA, SAL_HB6194AA, SAL_HB6195AA, SAL_HB6201AA, SAL_HB6207AA, SAL_HB6208AA, SAL_HB6213AA, SAL_HB6217AA, SAL_HB6232AA, SAL_HB6235AA, SAL_HB6236AA, SAL_HB6238AA, SAL_HB6239AA, SAL_HB6240AA, SAL_HB6242AA, SAL_HB6244AA, SAL_HB6261AA, SAL_HB5975AA. Data has been available in the database since April 2021.

## Author contributions

MC and MF conceptualized the study. MC performed the laboratory analysis and wrote the original draft. MC, BD'A, and RV performed data selection and data analysis. MC, BD'A, RV, and MF contributed to writing, proofreading, and editing. All authors contributed to the article and approved the submitted version.
