# Supplementary material for: Phenotypic and genotypic survey of antibiotic resistance in Salmonella enterica isolates from dairy farms in Uruguay
Source: Front Vet Sci. 2023 Mar 9;10:1055432. doi: 10.3389/fvets.2023.1055432 (PMC10033963; doi:10.3389/fvets.2023.1055432)
Supplement: Supplementary file 2 [file Table_2.DOCX]

| **Supplementary Table 2.**  **Genotypic and phenotypic resistance to antibiotics in *Salmonella enterica* isolates from dairy farms in Uruguay** | | | | | | | |
| --- | --- | --- | --- | --- | --- | --- | --- |
| **Sample** | **Isolate ID** | **Serotype** | **Phenotype**  **Not susceptible** | **Group of antibiotics** | **Number**  **of strains** | **Genotype resistance** | **Mutation** |
| 1 | 39195 39250 | **SDu** | CIP | FQ | 2 | *qnrB*19^(1)^ –*aac(6')-Iaa* | - |
| 2 | 39207 |  | S | AMG | 1 | *aac(6')-Iaa* | - |
| 3 | 39213 |  | TE | TE | 1 | *aac(6')-Iaa* | - |
| 4 | 39251 |  | CIP-S | FQ-AMG | 1 | *aac(6')-Iaa* | - |
| 5 | 39257 |  | CIP-ENR- AZM | FQ-MCL | 1 | *aac(6')-Iaa* | *acrB*_R717Q |
| 6 | 39190 | **SAn** | S | AMG | 1 | *aac(6')-Iaa* | *parC_*T57S |
| 7 | 39191  39198 39202  39216 39222  39225 39255 |  | ^(a)^CIP-S^(6)^  ^(b)^CIP-S-CN^(1)^ | FQ-AMG | 7 | *aac(6')-Iaa*-    (*aph(3”)-Ib*- *aph(6)-Id*-  *bla*TEM-1B-  *qacE*-  *dfrA*7-  *sul*1- *sul*2- *tet*(A)) *^(1)^* | *parC_*T57S |
| 8 | 39211  39221 |  | CIP-S-NF | FQ-AMG-NF | 2 | *aac(6')-Iaa* | *parC_*T57S |
| 9 | 39215 |  | AMP-SXT-CIP- S- TE | BL-SF-FQ-AMG-TE | 1 | *aac(6')-Iaa- aph(3”)-Ib- aph(6)-Id-  blaTEM-1B-  qacE-  dfrA7-  sul1- sul2- tet(A)* | *parC_*T57S |
| 10 | 39185 | **SNe** | S | AMG | 1 | *aac(6')-Iaa- aph(3”)-Ib- aph(6)-Id- tet(A)* | *parC_*T57S |
| 11 | 39230 |  | CIP | FQ | 1 | *aac(6')-Iaa* | *parC_*T57S |
| 12 | 39186 39194  39206  39237  39248  39256 |  | S-TE | AMG-TE | 6 | *aac(6')-Iaa-*  *aph(3'')-Ib- aph(6)-Id- tet(A)* | *parC_*T57S |
| 13 | 39183 |  | S-NF | AMG-NF | 1 | *aac(6')-Iaa* | *parC_*T57S |
| 13 | 39197 |  | CIP- S | FQ-AMG | 1 | *aac(6')-Iaa -*  *aph(3'')-Ib- aph(6)-Id- tet(A)* | *parC_*T57S |
| 15 | 39200 |  | CIP-ENR S-TE | FQ-AMG-TE | 1 | *aac(6')-Iaa-*  *aph(3'')-Ib- aph(6)-Id- tet(A)* | *parC_*T57S |
| 16 | 39177 39193  39217 39220  39239 39240 |  | ^(a)^CIP-S-CN- TE^(1)^  ^(b)^CIP-ENR-S-CN-TE^(1)^  ^(c)^CIP-S-TE^(4)^ | FQ-AMG-TE | 6 | *aac(6')-Iaa- aph(3'')-Ib- aph(6)-Id- tet(A)* | *parC_*T57S |
| 17 | 39180 |  | AMP-CIP-S-TE | BL-FQ-AMG-TE | 1 | *aac(6')-Iaa-*  *aph(3'')-Ib- aph(6)-Id- tet(A)* | *parC_*T57S |
| 18 | 39181 |  | AMC-AMP-CIP-ENR-S-CN-TE-NF | BL-FQ-AMG-TE- NF | 1 | *aac(6')-Iaa-*  *aph(3'')-Ib- aph(6)-Id- tet(A)* | *parC*_T57S |
| 19 | 39182 39236 |  | ^(a)^CIP- S- CN- TE- AZM  ^(b)^CIP-S-TE-AZM | FQ-AMG-TE-MCL | 2 | *aac(6')-Iaa-*  *aph(3'')-Ib- aph(6)-Id- tet(A)* | *parC*_T57S |
| 20 | 39231 |  | CIP-S-TE- NF | FQ-AMG-NF | 1 | *aac(6')-Iaa* | *parC*_T57S |
| 21 | 39208 |  | S-TE-NF | AMG-TE-NF | 1 | *aac(6')-Iaa-*  *aph(3'')-Ib- aph(6)-Id- tet(A)* | *parC_*T57S |
| 22 | 39209 |  | TE-AZM | TE-MCL | 1 | *aac(6')-Iaa-*  *aph(3'')-Ib- aph(6)-Id- tet(A)* | *parC_*T57S |
| 23 | 39212  39219  39238 | **STy** | CIP | FQ | 3 | *aac(6')-Iaa-*  *aph(3'')-Ib^(1)^- aph(6)-Id^(1)^- sul2^(1)^- tet(A)^(1)^-floR^(1)^* | - |
| 24 | 39189  39223 |  | S-TE | AMG- TE | 2 | *aac(6')-Iaa-*  *aph(3'')-Ib- aph(6)-Id- sul2- tet(A)* | - |
| 25 | 39201 |  | CIP-TE | FQ-TE | 1 | *aac(6')-Iaa-*  *aph(3'')-Ib- aph(6)-Id- sul2- tet(A)* | - |
| 26 | 39179  39192  39210  39214 39218  39232  39234 39243  39244 39252 |  | CIP-S-TE | FQ-AMG-TE | 10 | *aac(6')-Iaa-  aadA^(1)^-  aadA2^(1)^- aph(3')-Ia^(1)^ –*  *aph(3'')-Ib^(8)^- aph(6)-Id^(8)^- blaTEM-1B^(1)^- sul2 ^(8)^- floR^(1)^- cmlA1^(1)^-*  *qnrB19^(1)^- qacE^(1)^-dfrA12^(1)^-InuF^(1)^- tet(A)^(7)^- tet(M)^(1)^* | *-* |
| 27 | 39226 |  | S- TE- NF | AMG-TE-NF | 1 | *aac(6')-Iaa- aph(3'')-Ib- aph(6)-Id- sul2- tet(A)* | - |
| 28 | 39178  39187  39203  39228 39233 |  | ^(a)^CIP-S-TE-NF^(4)^  ^(b)^CIP-ENR-S-TE-NF^(1)^ | FQ-AMG-TE-NF | 5 | *aac(6')-Iaa- aph(3'')-Ib- aph(6)-Id- sul2- tet(A)^(4)^-qnrB19^(1)^* | - |
| 29 | 39199 |  | CIP-S-TE-NF-AZM | FQ-AMG-TE-NF-MCL | 1 | *aac(6')-Iaa- aph(3'')-Ib- aph(6)-Id- sul2- tet(A)* | - |
| 30 | 39229  39245  39249 |  | ^(a)^AMP-AMC-CIP-ENR-S-TE  ^(b)^AMP-CIP-S-TE  ^(c)^AMP-AMC-CIP-S-TE | BL-FQ-AMG-TE | 3 | *aac(6')-Iaa- aph(3'')-Ib- aph(6)-Id- blaTEM-1B- sul2 - tet(B)- aph(3')-Ia^(2)^- qnrB19^(1)^-* | - |
| 31 | 39235 |  | SXT-CHL-S-TE | S-F-AMG-TE | 1 | *aac(6')-Iaa- aadA- aadA2- aadA17- aph(3')-Ia- aph(3'')-Ib- aph(6)-Id- floR- cmlA1- qacE- dfrA12-lnuF-sul2- tet(A)- tet(M) -* | - |
| 32 | 39242 |  | AMC-CIP-S | BL-FQ-AMG | 1 | *aac(6')-Iaa- aph(3'')-Ib- aph(6)-Id- sul2- tet(A)* | - |
| 33 | 39246 |  | AMP-SXT-CIP- S- CN- TE-AZM | BL- S- FQ- TE-AMG- MCL | 1 | *aac(6')-Iaa- aph(3'')-Ib- aph(6)-Id- sul2- tet(A)* | - |
| 34 | 39253 |  | AMP-CIP- S- CN- TE-AZM | BL- S- FQ- TE-AMG- MCL | 1 | *aac(6')-Iaa- aph(3'')-Ib- aph(6)-Id- qnrB19-sul2- tet(A)* | - |
| 35 | 39254 |  | AMP-S-TE-NF | AMP-S-TE-NF | 1 | *aac(6')-Iaa- aph(3')-Ia- aph(3'')-Ib- aph(6)-Id- blaTEM-1B- sul2 - tet(B)* | - |
| 36 | 39184 | IIIB 61:I:Z53 | CIP-S-TE-NF | FQ-AMG-TE-NF | 1 | *aac(6')-Iaa* | *parC*_T57S |
| 37 | 39196 | SAg | TE | TE | 1 | *fosA7- tet(A)- aac(6')-Iaa* | *parC_T57S* |
| 38 | 39224 | SMo | CIP-S | FQ- AMG | 1 | *aac(6')-Iaa- fosA7* | *parC_T57S* |

References: STy: *S.* Typhimurium; SNe: *S.* Newport; SAn: *S.* Anatum; SDu: *S.* Dublin; SMo: *S.* Montevideo; SAg: *S.* Agona. AMC: amoxicillin-clavulanic acid; AMP: ampicillin; CTX: cefotaxime; SXT: trimethoprim-sulfamethoxazole; CIP: ciprofloxacin; ENR: enrofloxacin; S: streptomycin; CN: gentamicin; TE: tetracycline; CHL: chloramphenicol; NF: nitrofurantoin. AMG: aminoglycosides; BL: beta lactams; F: phenicol; FQ: quinolones; S: trimethoprim-sulfonamides; TE: tetracycline; MCL: azithromycin; NF: nitrofurans. Numbers in parentheses: number of isolates presenting the gene/mutant.
